# Unanticipated Large-Scale Deletion in *Fusarium graminearum* Genome Using CRISPR/Cas9 and Its Impact on Growth and Virulence

**DOI:** 10.3390/jof9060673

**Published:** 2023-06-14

**Authors:** Adam John Foster, Emily Johnstone, Abbey Saunders, Eva Colic, Nicole Lassel, Janesse Holmes

**Affiliations:** 1 Charlottetown Research and Development Centre, Agriculture and Agri-Food Canada, Charlottetown, PE C1A 4N6, Canada; 2 Summerland Research and Development Centre, Agriculture and Agri-Food Canada, Summerland, BC V0H 1Z0, Canada

**Keywords:** *Fusarium graminearum*, CRISPR/Cas9 editing, large deletion, dual ribozyme sgRNA processing

## Abstract

*Fusarium graminearum*, a filamentous fungus, and causal agent of Fusarium head blight (FHB) in wheat and other cereals, leads to significant economic losses globally. This study aimed to investigate the roles of specific genes in *F. graminearum* virulence using CRISPR/Cas9-mediated gene deletions. Illumina sequencing was used to characterize the genomic changes due to editing. Unexpectedly, a large-scale deletion of 525,223 base pairs on chromosome 2, comprising over 222 genes, occurred in two isolates. Many of the deleted genes were predicted to be involved in essential molecular functions, such as oxidoreductase activity, transmembrane transporter activity, hydrolase activity, as well as biological processes, such as carbohydrate metabolism and transmembrane transport. Despite the substantial loss of genetic material, the mutant isolate exhibited normal growth rates and virulence on wheat under most conditions. However, growth rates were significantly reduced under high temperatures and on some media. Additionally, wheat inoculation assays using clip dipping, seed inoculation, and head point inoculation methods were performed. No significant differences in virulence were observed, suggesting that these genes were not involved in infection or alternative compensatory pathways, and allow the fungi to maintain pathogenicity despite the extensive genomic deletion.

## 1. Introduction

*Fusarium graminearum Schawbe* (teleomorph *Gibberella zeae* (Schw.) Petch), is a filamentous fungus responsible for Fusarium head blight (FHB) in wheat and other small grain cereals, as well as diseases in other crops, causing substantial economic losses worldwide [1,2]. Understanding the biology and virulence factors of this devastating pathogen is crucial for developing effective strategies to combat crop diseases.

In recent years, clustered regularly interspaced short palindromic repeats (CRISPR/Cas9) gene editing technology has revolutionized molecular biology and holds great promise in various applications, ranging from agriculture to medicine [3,4]. However, concerns have been raised regarding the off-target or unanticipated effects of CRISPR/Cas9 editing, which may lead to unintended consequences in organisms [5]. This issue becomes particularly important when considering its use in non-model organisms like filamentous fungi [6]. Off-target effects can lead to undesired mutations and functional changes, which could impact research discoveries [7].

Accurate gene editing is crucial for understanding fungal pathogenicity, virulence, and developing strategies to mitigate crop diseases. Minimizing off-target effects in CRISPR/Cas9 editing for filamentous fungi research involves rigorous protocols and methods for sgRNA design, target selection, and screening [8,9,10]. The use of transient ribonucleoproteins can also lead to efficient CRISPR editing [11]. Intrinsic dual ribozyme sgRNA designs have shown success in filamentous fungi, allowing for self-processed RNA cleavage and the liberation of sgRNA from a transcript driven by a strong Polymerase II promoter [12,13]. Despite rigorous protocols and meticulous sgRNA design, unanticipated large deletions have been reported at on-target sites during gene editing. These unexpected mutations have been observed in diverse species, including human progenitor and stem cells, mouse embryos, and the filamentous fungi *Magnaporthe oryzae*, with deletion sizes ranging from hundreds to thousands of base pairs [14,15,16,17]. These unexpected mutations underline the necessity of thoroughly examining on-target insertions and deletions due to their potential to greatly influence the outcome of CRISPR gene editing [18].

In the current study, we describe an attempt at targeted deletions of three genes using CRISPR/Cas9 in *F. graminearum* in an effort to characterize their role in virulence on wheat (*Triticum aestivum* L.) while incorporating techniques for accurate sgRNA targeting. However, instead of the intended specific deletions, we observed an unexpected large-scale deletion of over 500,000 base pairs (bp) on chromosome 2 at an on-target site. This unintended deletion led to the loss of over 222 different genes in the affected region. Despite the significant alteration in the genome, the mutant isolate demonstrated mostly normal growth rates and maintained its virulence on wheat. This outcome highlights the importance of rigorous experimental design and validation when using CRISPR/Cas9 in filamentous fungi because unintended genomic alterations can occur. Without deep genomic sequencing, the extent of the deletion observed in some isolates in this study may not have been discovered.

## 2. Materials and Methods

### 2.1. Identification of Initial F. graminearum Genes for Targeted Deletion and Disruption Strategy

All experiments were conducted using the wild-type *F. graminearum* isolate DAOMC221140 (wt) obtained from the Canadian Collection of Fungal Cultures. To identify genes potentially associated with FHB virulence, we analyzed the *F. graminearum* PH-1 (RR1) genome (Ensembl Fungi release 56; https://fungi.ensembl.org/Fusarium_graminearum, accessed on 24 April 2020) for unannotated genes encoding putative small secreted cysteine rich proteins (SSCRPs) [19]. Three target genes were selected based on not being previously characterized, their similarity in encoded amino acid sequences, and that the genes were not clustered, so that a CRISPR gene editing technique would be an advantageous approach to gene editing. The gene IDs for these targets are FGSG 03445 (FGRAMPH1 01G12903), FGSG 08238 (FGRAMPH1 01G09441), and FGSG 04583 (FGRAMPH1 01G15633) [20]. All three genes are located on chromosome 2 but are distributed across a wide range, with FGSG 08238 at 781,898 bp, FGSG 03445 at 5,178,101 bp, and FGSG 04583 at 8,472,232 bp. After aligning the amino acid sequences using ClustalW, the three genes exhibited 55.4% to 71.6% amino acid identity. The close genetic relationship between the genes provided an ideal platform for evaluating CRISPR/Cas9 performance because two of the genes shared CRISPR target sites.

A gene editing strategy was devised to disrupt the coding sequences of the selected SSCRPs encoding genes. A plasmid vector, complete with a codon-optimized Cas9 gene and numerous dual ribozyme-flanked sgRNAs for accurate processing, was constructed. Subsequently, a PEG-mediated protoplast transformation was executed. Variable numbers of sgRNAs were designed to target each gene for evaluating the resultant mutations. Verification of transformations and editing events ensued via polymerase chain reaction (PCR) and genome sequencing.

### 2.2. sgRNA Design for Target Genes

To design CRISPR/Cas9 target sequences for the target genes, we employed the CRISPOR web tool [21] using the *F. graminearum* PH-1 (GZPH1RRResV1) genome as a reference. Our gene disruption strategy aimed to disrupt approximately 100 bp of the coding region by targeting one to three sgRNA sequences at each gene. We designed a total of five sgRNAs, summarized in Table 1. FGSG 04583 was targeted with a single sgRNA.

### 2.3. Vector Construct for CRISPR Editing

The vector pEGC-sGR1 was constructed from the vector backbone pFGL821 (Addgene plasmid # 58223; http://n2t.net/addgene:58223; RRID: Addgene_58223), inspired by the strategy outlined by Gardiner and Kazan [13]. Four separate DNA cassettes were synthesized to produce the expression vector:

1. GPDA_exp_cassette: Containing the gpdA promoter from *Aspergillus nidulans*, a 20 bp spacer with the AscI cut site, and the *F. graminearum* Tri4 terminator, synthesized by GeneArt (Life Technologies Inc., Toronto, ON, Canada).
2. Cas9-2018ABP2XP: A *F. graminearum* codon-optimized gene encoding the native Cas9 protein, also synthesized by GeneArt synthetic gene synthesis (Life Technologies Inc., Canada).
3. RNA_exp_cassette: Comprising five sgRNAs embedded between hepatitis delta virus (HDV) and hammerhead ribozymes, followed by an enhanced green fluorescent protein (GFP)-encoding marker gene. The expression of the sgRNA is driven by the *A. nidulans* translation elongation factor promoter (pTef1) and followed by the *A. nidulans* trpC terminator. This cassette was synthesized by General Biosystems (Duram, NC, USA).

**Figure 1:**
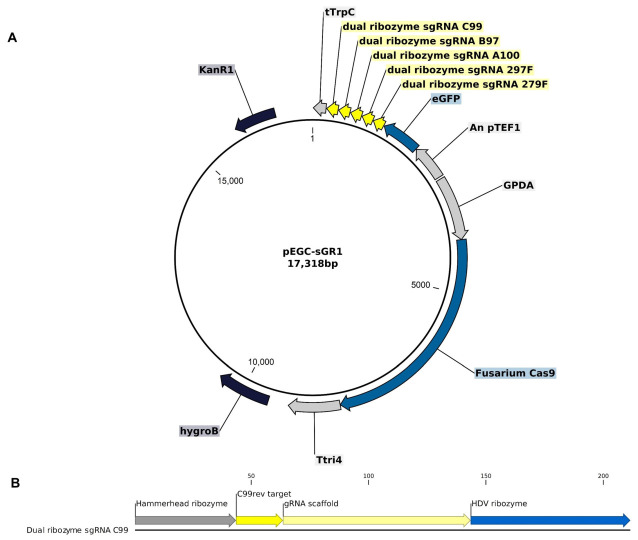
Vector for CRISPR/Cas9 gene disruption of *F. graminearum*. (**A**) pEGC-sGR1 plasmid derived from pFGL821, featuring hygromycin B resistance, Cas9 gene with codon optimization driven by GPDA promoter, and a cassette encoding 5 sgRNAs, flanked by dual ribozymes, and codon-optimized GFP gene driven by the pTef1 promoter. (**B**) Example sgRNA cassette flanked by 5′ and 3′ sequences encoding a hammerhead ribozyme and HDV ribozyme, respectively, to process each sgRNA cassette.

Vector sequences were digested with appropriate restriction enzyme and standard cloning techniques. Anza restriction enzymes (Thermo Scientific, Mississauga, ON, Canada) were used for digestions, T4 DNA Ligase (Invitrogen Canada Inc., Burlington, ON, Canada) was utilized for inserting cassettes into the vector, *E. coli* transformations were conducted using chemical heat-shock methods with competent NEB 5-alpha cells (New England Biolabs, Ipswich, MA, USA), and QIAprep Spin Miniprep Kit (Qiagen, Germantown, MD, USA) was used for plasmid purifications. All procedures were conducted using manufacturers’ instructions. Three separate assembly reactions were performed to generate the final p-EGC-sGR1 (Figure 1). First, the GPDA_exp_cassette was cloned into vector pFLG821 at the XbaI site to produce a new purified vector, then Cas9-2018ABP2XP was cloned into this vector by digesting and ligating into the SgsI and PacI sites in the GPDA_exp_cassette. Finally, the gRNA_exp_cassette was cloned into the EcoRI site of the multicloning site of the previous step’s vector product. The integrity of the assembled vector was confirmed through restriction digest analysis and Sanger DNA sequencing by Eurofins Genomics (Toronto, ON, Canada). The sequence and annotation for pEGC-sGR1 are included in Supplementary File S1.

### 2.4. Protoplast Transformation

Protoplast transformation was performed as described by Desmond et al. [22] and Guillemette et al. [23] with modifications. Solutions for protoplast transformation are listed in Table S1. *Fusarium graminearum* wt was grown from a single mycelial plug on potato dextrose agar (PDA; BD Difco, Franklin Lakes, NJ, USA) at 28 °C for three to five days. Three 2.5 mm plugs of mycelium from the culture’s edge were transferred to a 250 mL tissue culture flask containing 40 mL of potato dextrose broth (PDB; BD Difco, Franklin Lakes, NJ, USA), and incubated at room temperature for two days, then the contents of the flask were transferred into a 50 mL tube and centrifuged for 10 min at 4500 RCF. The supernatant was removed, and the pellet was resuspended in 30 mL of protoplasting buffer. The mycelium was collected in a 40 µM basket filter and transferred to a 50 mL cell culture flask. Freshly prepared protoplasting solution was added to the flask and incubated overnight at 33 °C with gentle shaking to generate protoplasts.

After overnight digestion, the protoplasts were washed with 30 mL of W5, mixed by inverting the tube three to five times. The mixture was then poured through a 40 µM basket filter into a 50 mL tube to separate undigested mycelium and cellular debris from the protoplasts. The tube was centrifuged at 4500 RCF and 4 °C for five min to pellet the cells, which were then resuspended in W5. Aliquots of resuspended protoplasts were enumerated on a haemocytometer. Then, protoplasts were resuspended in the residual W5 to a final concentration of 1 × 10^6^ mL^−1^. For transformation, 100 µL of protoplast solution was used. Next, 25 µL of plasmid DNA (5 µg pEGC-sGR1) was added to a 15 mL tube and incubated on ice for 10 min. Then, 200 µL of ice-cold 40% PEG-Calcium solution was added and incubated for an additional 10 min on ice. This step was repeated. Finally, 800 µL of 40% PEG-Calcium was added and incubated at room temperature for 10 min. To stop the reaction, 1 mL of room temperature W5 was added. The contents of Flasks A, B, and C were combined into a recovery media, cooled to approximately 55 °C, then protoplasts were added, gently mixed, and poured onto eight 100 mm Petri-dishes. Plates were incubated in the dark at 28 °C for 24 hrs. To select *F. graminearum* transformants, a 10 mL antibiotic overlay of 1% water agar amended with 150 mg mL^−1^ of hygromycin B (Gibco 10687010, Themo Fisher, Mississauga, ON, Canada) was added. Colonies that grew on the overlay up to two weeks post-transformation were subcultured on PDA amended with 50 mg mL^−1^ of hygromycin B and grown for three to five days. DNA was extracted from these transformed isolates by scraping small amounts of hyphae into 2 mL microcentrifuge tubes containing 200 µL of TE buffer pH 7.5 and approximately 10 mg of acid-washed sand. Mycelium was disturbed by vortexing for 30 s. This crude extract was used as a template for PCR using Phusion Hot Start II High-Fidelity PCR Master Mix and target gene primers (Table 2) in standard reactions where after an initial denaturation at 98 °C for 2 min, the temperature was cycled through 98 °C for 10 s, 60 °C for 20 s, and 72 °C for 15 s for 35 cycles. PCR products were evaluated in 1% agarose gels amended with SybrSafe DNA gel stain (Thermo Fisher, Mississauga, ON, Canada) and visualized using a blue light E-Gel Imager (Thermo Fisher, Mississauga, ON, Canada). Reduction in band sizes compared to wt was initially used to confirm the presence of CRISPR/Cas9 edits. Positive transformants were also screened by fluorescent microscopy for the presence of GFP in hyphae.

### 2.5. Genome Sequencing of F. graminearum Isolates to Investigate Gene Disruption

Transformed isolates Tk-1 and Tk-3 showed expected disruptions in genes FGSG 03445 and FGSG 08238; however, disruption could not be confirmed for gene FGSG 04583. To further investigate this, genome sequencing was performed on four isolates: Tk-1, Tk-3, Tk-19, and the wt isolate. Isolates were grown in 50 mL of PDB at 28 °C for three days. Hyphae were collected by filtering through 100 µm basket filters and washed three times with sterile water to remove residual media. High molecular weight DNA was extracted using the NucleoBond HMW DNA kit (Macherey-Nagel GmbH & Co. KG, Düren, Germany) following the manufacturer’s protocol. Mycelium was disrupted by grinding in a mortar and pestle in liquid nitrogen. DNA samples were sequenced using the Illumina NovaSeq (PE150) platform by Novogene Co. (Sacramento, CA, USA). Raw reads were quality trimmed and mapped to the *F. graminearum* PH-1 reference genome using CLC Genomics Workbench 20 (Qiagen, Germantown, MD, USA) with default Illumina trimming settings. This approach allowed for the detailed investigation of gene disruption events in the selected isolates.

### 2.6. Growth Rate Assessment of F. graminearum Isolates

Growth rates of the wt and Tk-1 were tested on rich medias including PDA, yeast mold (YM) (BD Difco, Franklin Lakes, NJ, USA), oatmeal (Sigma-Aldrich, St. Louis, MO, USA), and semi-synthetic Czapek-Dox (CZ; Sigma-Aldrich) agar media at 20 °C. Additionally, isolates were grown on PDA at temperatures of 10, 20, 30, and 40 °C. The growth rate experiments were conducted by inoculating plates with the respective isolates and measuring colony diameter after 94 hr. Differences in growth rate between wt, Tk-1, and Tk-3 were determined using analysis of variance (ANOVA), Tukey’s HSD test, and Students *t*-test in JMP 17.0 (SAS Institute, Cary, NC, USA). This approach allowed for the comparison of growth characteristics between the isolates.

### 2.7. Effect of Gene Disruption on F. graminearum Virulence of Wheat

Three experiments were conducted to evaluate the variation in virulence among *F. graminearum* isolates on the wheat cultivar AC Walton. This cultivar is a popular registered milling variety in Atlantic Canada, and exhibits moderate susceptibility to *F. graminearum*. These experiments aimed to detect differences in root disease, leaf disease, and FHB virulence. Each experiment was performed with four replications.

1. Root disease assay: Wheat seeds were planted in Pro-Mix soil-filled 50 mL tubes and inoculated with a suspension of mycelium. The mycelium was cultivated in PDB liquid media for a week at 25 °C, mildly shaken at 50 RPM, centrifuged at 3200 CFU for 5 min, and the resultant mycelial pellet was resuspended in RO water. After disrupting the mycelium in a blender, the suspension was normalized to 200,000 CFU mL^−1^. Each seed was treated with 500 µL of the disrupted isolate, and seedling germination, emergence, and visual disease were documented at 3, 7, and 14 days after inoculation DAI.
2. Clip dipping assay: Clip dipping protocol was modified from Shin et al. [24]. Wheat plants were grown in 50 mL tubes in Pro-Mix soil with four seeds per pot until they reached the Feekes 1.3 growth stage with three expanded leaves. Leaves were trimmed with scissors and dipped into a suspension of disrupted mycelium, produced as described above. Measurements of disease progression were taken at 3 and 7 DAI.
3. Modified wheat point inoculation assay: This assay was modified from Feng and Tang [25]. Wheat plants were grown in a 1:1 mixture of sterilized field soil and Pro-Mix in 7.5-L pots until they reached anthesis, with 200 mL per pot of 1/4 Hoagland’s solution supplied weekly. The fifth anther from the base of the seed head was inoculated with a 2 mm plug of hyphae taken from the edge of a five-day-old culture grown on PDA at 25 °C. Plugs were attached with parafilm, and the entire plant was bagged to the base of the pot for 48 h to allow for disease development. Bags were removed, and disease was rated at 7 and 14 DAI by counting the number of infected spikelets showing bleached symptoms. A selection of diseased wheat tissues were surface sterilized with 70% ethanol and plated on PDA to confirm *F. graminearum* colonization.

The root disease and clip dipping experiments were conducted in a Conviron growth chamber at 25 °C and 80% humidity with a 16-hr light cycle. The modified wheat point inoculation experiment was conducted in a greenhouse at 24 °C with 16 hr supplementary light. In all the trials, virulence differences between wt and transformed isolates were determined using ANOVA and Tukey’s HSD test in JMP 17 (SAS Institute, Cary, NC, USA).

### 2.8. Characterization of Mutant Isolates and Functional Analysis of Deleted Genes

To assess the potential functional consequences of the loss of the 222 predicted genes disrupted in the deletion of chromosome 2 in two mutant isolates, the gene sequences were analyzed using BLAST2GO software (BioBam Bioinformatics, Valencia, Spain).

## 3. Results and Discussion

**Figure 2:**
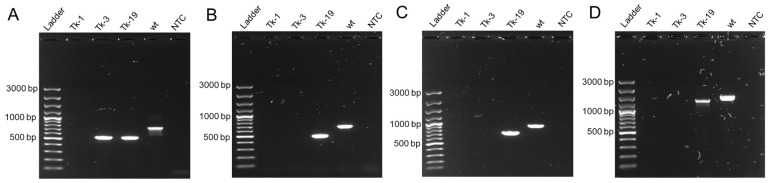
Agarose gel (1%) showing PCR-amplified region of disrupted genes in transformed isolates. Ladder = ThermoScientific GeneRuler 100 bp Plus DNA ladder (Cat. No. SM0321). PCR products for (**A**) FGSG 03445 amplified by 4A-17-F and 4A-756-R, (**B**) FGSG 08238, amplified by 4B-9-F and 4B-673-R, (**C**) FGSG 04583, amplified by 4C-23-F and 4C-962-R, and (**D**) FGSG 04583, amplified by 4C-Fl-F and 4C-Fl-R. NTC = not template control reaction.

Three independent protoplast transformations were conducted, and a total of 19 transformed subcultures were obtained. The presence of GFP was confirmed in 17 of 19 transformed isolates by fluorescent microscopy (Figure S1). A subset of isolates were screened by PCR using primers for the three target genes. PCR successfully amplified products of reduced size for all target genes for isolate Tk-19, indicating all target genes had deletions of ~100–200 bp (Figure 2). Two isolates did not produce PCR products. Isolate Tk-1 failed to produce a product for any primer set, while Tk-3 only yielded a product for gene FGSC 03445, which indicated a deletion of ~200 bp compared to wt. Additional reactions were performed with primers flanking gene FGSG 04583, listed in Table 2, but again, no bands were visible to Tk-1 or Tk-3 (Figure 2).

**Figure 3:**
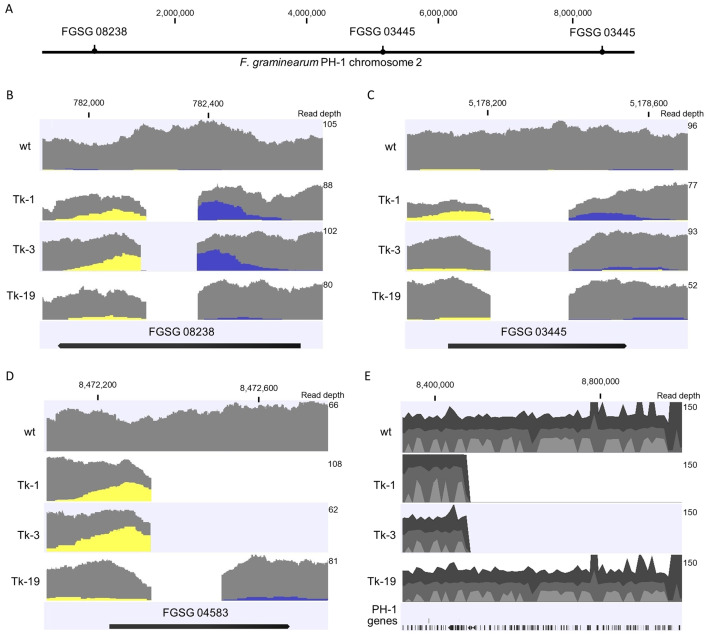
Location of target genes (**A**) and mapping depth of reads to genes (**B**) FGSG 08238, (**C**) FGSG 03445, and (**D**) FGSG 04583 in the *F. graminearum* PH-1 genome. Yellow indicates left single-mapped reads, grey indicated paired-mapped reads, and blue indicates right single-mapped reads. (**E**) Extent of deletion to chromosome 2, where dark grey indicates forward paired-mapped reads, medium grey indicates reverse paired-mapped reads, and light grey indicates proportion of single-mapped reads. Insertion of the transformation vector was detected in FGSG 08238 for Tk-1 and Tk-3, FGSG 03445 for Tk-1, and FGSG 04583 for Tk-1 and Tk-3 at the sgRNA cut sites as pairs of the single-mapped reads matched regions of pEGC-sGR1. Maximum read depth of the selected region is indicated on the right axis.

To investigate these isolates further, Illumina Nova Seq sequencing was conducted on four isolates, including the wt and the two with unconfirmed deletion of gene FGSG 04583. The reads were mapped to the *F. graminearum* PH-1 genome. A summary of DNA sequencing results and mapping statistics is in Table 3, and each genome was mapped to a median read depth of 55–72×. Isolate Tk-19 was found to have successful deletion of all target genes with deletion sites matching those estimated by PCR (Figure 3). In addition, sequencing results confirmed the successful and expected deletion of FGSG 03445 and FGSG 08238 with cuts at the target sites for Tk-1 and Tk-3 (Figure 3B,C). However, insertion of the vector was detected at the cut sites for Tk-1 for both genes and Tk-3 for gene FGSG 08238. Approximately 20–80 bp of the reads mapped at the cut sites overlapped to the vector sequence, but the exact size for the insertion could not be determined. This corresponds with the failed PCR reactions for these target genes and may indicate that the insertion of vector DNA was too large to be detected in our PCR assay.

**Figure 4:**
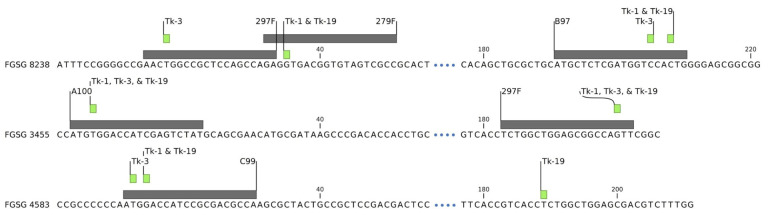
Summary of observed deletion sites on target genes in isolates Tk-1, Tk-3, and Tk-19. sgRNA target sites (grey) and detected cut sites (green) in CRISPR edited isolates Tk-1, Tk-3, and Tk-19. All isolates showed the same cut sites for gene FGSG 3455. Tk-1 and Tk-19 had the same cut sites in FGSG 8238, but Tk-3 was cut at the 297F target sites instead of 279F and had four less bp deleted at the B97 site. Tk-1 and Tk-19 were both cut at the expected C99 cut site, but Tk-3 had three more bp deleted in FGSG 4583. Tk-19 was also cut 175 bp from the C99. Location on chromosome 2 of the first base in the selection is indicated below the gene name. Sequence is split by dots for readability to indicate the nontargeted space between 5′ and 3′ sgRNA sites.

Gene FGSG 04583 was targeted with only a single sgRNA, and the unexpected deletions occurred at this site. Tk-1 and Tk-3 both had cut sites at the target site at 8,472,335 of chromosome 2, but no reads aligned after this, indicating a total deletion of the end of the chromosome after this site (Figure 3D,E). The observed loss of 525,223 bp resulted in the deletion of 222 confirmed or hypothetical genes. Genome sequencing results, as illustrated in Figure 4, verified the deletion sites for each gene. Notably, Tk-3 displayed distinct specific cut sites for FGSG 08238 and FGSG 04583 compared to Tk-1 and Tk-19. Tk-19 was also cut 175 bp from the C99 target site, possibly through non-homologous end joining (NHEJ) repair errors [4]. Other mechanisms have been hypothesized for large unanticipated CRISPR deletions including those that may arise from slower double-strand break repair processes, microhomology-mediated repair mechanisms, or the occurrence of single double-strand breaks in close proximity to target sites [15,17,18]. Furthermore, an overlap of the transformation vector at the large deletion site in Tk-1 and Tk-3 suggests this may be the site of vector insertion. It is also possible that the large deletion could have occurred through single-strand annealing repair at the cut site through insertion of the vector [18]; however, this hypothesis warrants further investigation.

**Figure 5:**
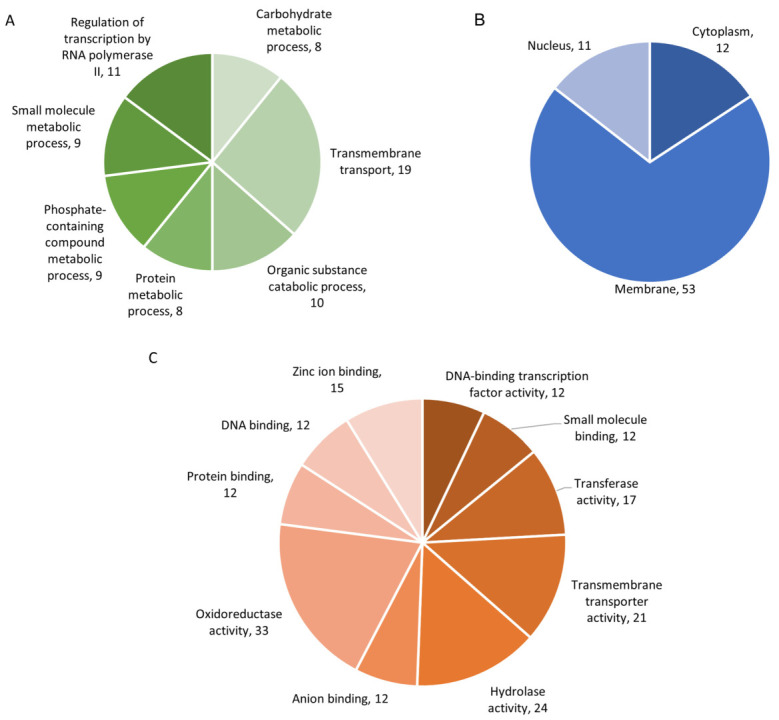
Analysis of disrupted genes by BLAST2GO. Multilevel summary pie charts summarize the genes’ (**A**) biological processes, (**B**) cellular components, and (**C**) molecular function. Numbers correspond to the quantity of genes predicted to each annotation. Some genes have multiple annotations.

The disrupted genes were analyzed for gene ontology using Blast2GO to determine cellular components, biological processes, and molecular functions (Figure 5; Table S2). The disrupted genes and their molecular functions play crucial roles in the host–pathogen interactions between *F. graminearum* and its plant hosts. Overall, 146 of the 222 genes (65.66%) were annotated. A total of 73 disrupted genes were annotated to biological processes, which we hypothesized might impact the fungi’s growth and virulence. These processes include carbohydrate metabolic processes, transmembrane transport, organic substance catabolic processes, and protein metabolic processes (Figure 5A). Altering the pathogen’s capacity to metabolize and transport nutrients may affect its growth and proliferation within the host, as efficient nutrient acquisition is essential for fungal virulence [26]. A total of 65 sequences were characterized to cellular components, with 54 being associated with the cell membrane, which plays a critical role in host–pathogen interactions (Figure 5B). The cell membrane serves as an interface between the pathogen and its host, allowing the exchange of molecules and signals essential for infection, growth, and virulence [27]. In addition to the cell membrane, other cellular components, such as the cytoplasm and nucleus, also play significant roles in the pathogen’s infection process because they house essential cellular machinery and genetic material involved in pathogenicity [28]. The majority of the disrupted genes (123 of 222) were identified with molecular functions that have been implicated in several processes, such as oxidoreductase activity, hydrolase activity, transmembrane transporter activity, transferase activity, DNA-binding transcription factor activity, small molecule binding, anion binding, protein binding, DNA binding, and zinc ion binding (Figure 5C). Many molecular functions previously investigated—such as transmembrane transporter activity, oxidoreductase activity, and small molecule binding—are directly linked to the cell membrane’s role in facilitating these processes [29].

There are several specific genes in the disrupted genes region of chromosome 2 that are widely understudied (Table S2). One example is FGRAMPH1 01G15715, which is predicted to encode a vegetative incompatibility protein HET-E-1, which may play a crucial role in fungal self/non-self-recognition and programmed cell death, which is essential for maintaining the genetic integrity and diversity of fungal populations [30]. Disruption of HET-E-1 in Tk-1 and Tk-3 may affect fungal development, colony formation, and overall fitness by compromising the vegetative incompatibility system. Additionally, the disruption of two genes, FGRAMPH1 01G15659 and FGRAMPH1 01G15663, putatively encoding a short chain dehydrogenase/reductase and an O-methyltransferase, respectively, may be involved in diterpenoid biosynthesis. Diterpenoids represent a diverse class of secondary metabolites known for their various biological activities, such as anti-inflammatory, antimicrobial, antifungal, and phytotoxic properties [31]. If the disruption of these genes results in the alteration or loss of specific diterpenoids, it could affect the production of secondary metabolites that contribute to the pathogen’s virulence or influence a range of other phenotypes.

**Figure 6:**
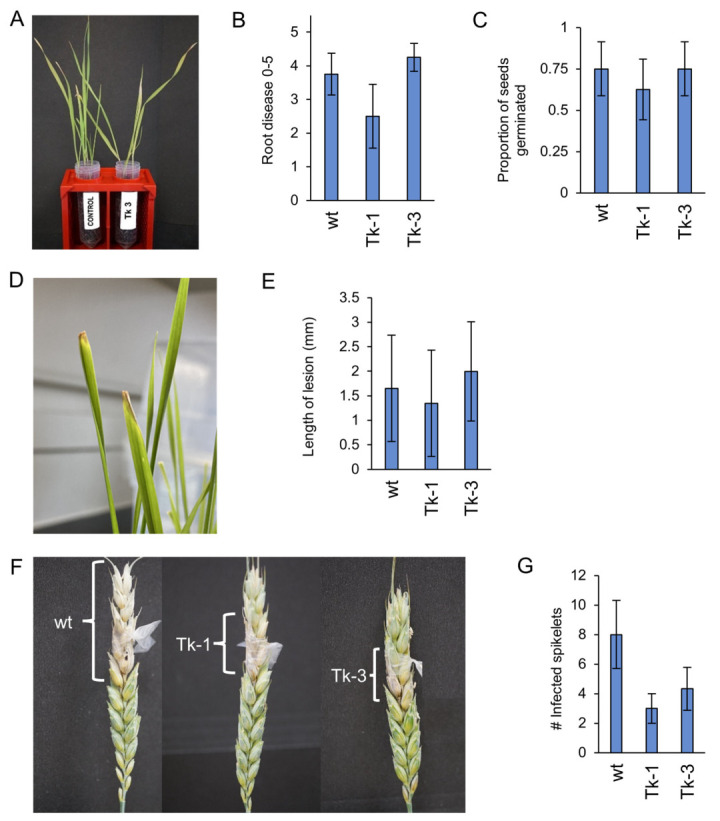
Comparative wheat virulence assessment of wt and mutated *F. graminearum* isolates. (**A**) Visual comparison of disease symptoms after seedling inoculation with isolate Tk-3 vs. untreated control, 14 days post-inoculation (DAI); (**B**) subjective root disease ratings; (**C**) proportion of germinated seeds following inoculation; (**D**) clip dipping leaf lesions on inoculated leaves; (**E**) lesion measurements, 7 DAI; (**F**) visibly infected spikes post-point inoculation; (**G**) total count of visibly infected spikelets, 14 DAI. Error bars represent standard errors. ANOVA and Tukey’s HSD test revealed no statistically significant differences between wt and mutated isolates.

Three experiments were conducted to assess the virulence of mutated isolates, Tk-1 and Tk-3, relative to the wt isolate. No statistically significant differences were observed for virulence in either the seedling (Figure 6A–C), clip dipping (Figure 6D,E), or modified point inoculation (Figure 6F,G) of wheat. These results indicate that the deleted genes might not have a significant impact on the virulence of *F*. *graminearum*, or that compensatory mechanisms may exist within the fungus to maintain its virulence. Alternatively, limitations in the experimental design or the specific conditions tested may have precluded the observation of phenotypic differences related to the deleted genes.

**Figure 7:**
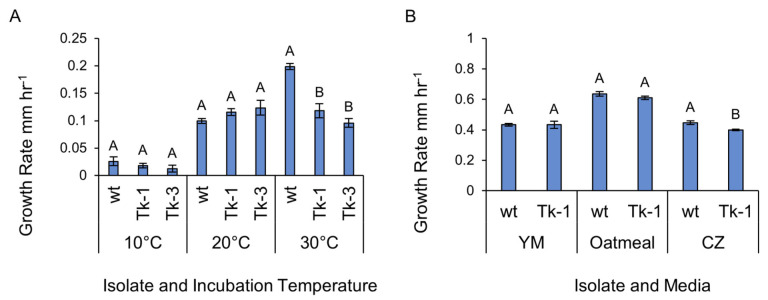
Growth rate characteristics of *F*. *graminearum* wt and mutated isolates. Growth rates of isolates grown at temperatures of 10, 20, and 30 °C grown on PDA. Vertical bars represent standard errors; bars sharing letters within temperatures are not significantly different at *α* ≤ 0.05 using Tukey’s HSD test (**A**). Growth rates of wt and mutant Tk-1 on YM agar, oatmeal agar, and CZ agar media. Vertical bars represent standard errors. Bars sharing letters within media are not significantly different at *α* ≤ 0.05 using Student’s *t*-test (**B**).

The hourly growth rate of *F. graminearum* wt and mutated Tk-1 and Tk-3 isolates was assessed at various incubation temperatures and on different media types. While no significant differences were observed among isolates grown at 10 °C and 20 °C on PDA, the wt displayed a significantly higher growth rate at 30 °C (Figure 7A). No growth was observed for any isolate at 40 °C. Furthermore, when comparing Tk-1 and the wt on YM, oatmeal, and CZ agar media, the wt showed a significantly higher growth rate on semi-synthetic CZ agar, but no differences were observed on non-synthetic YM or oatmeal agar (Figure 7B). The observed growth rate differences at 30 °C and on CZ agar could be attributed to the deletion of specific genes, which may affect the fungus’s ecological fitness or competitiveness under certain environmental conditions [32]. Further investigation is necessary to pinpoint the specific deleted genes responsible for these differences and examine their roles in *F. graminearum* biology, which may include stress response, secondary metabolism, and interactions with other microbes.

In conclusion, this study documents an abnormal effect of CRISPR/Cas9 editing *in F. graminearum*. The CRISPR/Cas9 system successfully enabled targeted editing of multiple genes in a single transformation event, in isolate Tk-19. However, a massive, unexpected deletion occurred, leading to the loss of 222 predicted genes in two other transformed isolates. Interestingly, none of these 222 genes were previously characterized as core metabolic genes, and the loss of such a significant number of genes did not result in noticeable changes in growth or virulence. Further investigation into the cause of the large deletion may provide valuable insights into the mechanisms of DNA double-strand break repair in *F. graminearum*, contributing to improved targeted editing in the future. To prevent such large deletions, employing two or more sgRNA to target genes may be a useful strategy.

## Figures and Tables

**Table 1:** sgRNA sequences and targets.

ID	Sequence	Target 1	Target 2
A100	ATAGACTCGATGGTCCACAT	FGSG 3455	--
B97	ATGCTCTCGATGGTCCACTG	FGSC 8238	--
C99	TGGCGTCGCGGATGGTCCAT	FGSC 4583	--
279F	GGCGACTACACCGTCACCTC	FGSC 8238	--
297F	TCTGGCTGGAGCGGCCAGTT	FGSG 3455	FGSC 8238

**Table 2:** List of primers to identify gene disruptions of target genes.

Target	ID	Primer Seq	Region Chromosome 2	wt Product Size (bp)
03445	4A-17-F	TACGCCTGGACCCTATCAAC	5,177,854–5,177,873	740
	4A-756-R	CCTGGTACTCAGAGCCCATC	5,178,574–5,178,593	
08238	4B-9-F	CAACTCAATCACTCGCTTCAA	782,571–782,591	665
	4B-673-R	CTCATTATGTATTGCCGCACA	782,851–782,870	
04583	4C-23-F	TCGTCTCTTTTCATCCTCATCA	8,472,131–8,472,152	940
	4C-962-R	TCCATCACTCTTTTGGGTGAG	8,473,050–8,473,070	
04583	4C-FL-F	CTGCGACATGCAGATGTACC	8,471,771–8,471,790	1498
	4C-FL-R	GGACACTGGGAGCAACTCTC	8,473,249–8,473,268	

**Table 3:** Genome resequencing and mapping results.

Isolate	Raw Reads	Reads after Trimming	Reads Mapped	Median Coverage
wt	19,013,264	19,011,008	97.01%	72×
Tk-1	14,778,038	14,776,430	96.83%	55×
Tk-3	18,104,336	18,102,091	96.75%	61×
Tk-19	16,428,990	16,427,116	96.27%	72×

## Data Availability

Data stored at Agriculture and Agri-food Canada may be made available on request.

## Supplementary Materials

The following supporting information can be downloaded at: https://www.mdpi.com/article/10.3390/jof9060673/s1, Table S1. Protoplast transformation media, buffers, and solutions modified from Guillemette et al. (2011). Table S2. Blast2GO annotations of the 222 disrupted genes. Figure S1. Bright field and fluorescent images of wt, Tk-1, Tk-3, and Tk-19. File S1. pEGC-sGR1.gbk (GenBank file for transformation vector). File S2. unedited gel images for Figure 2.
