# Supplementary material for: Unanticipated Large-Scale Deletion in Fusarium graminearum Genome Using CRISPR/Cas9 and Its Impact on Growth and Virulence
Source: J Fungi (Basel). 2023 Jun 14;9(6):673. doi: 10.3390/jof9060673 (PMC10303880; doi:10.3390/jof9060673)
Supplement: Supplementary file 1 [file jof-09-00673-s001.zip › Supplemental Figure S1.pdf]

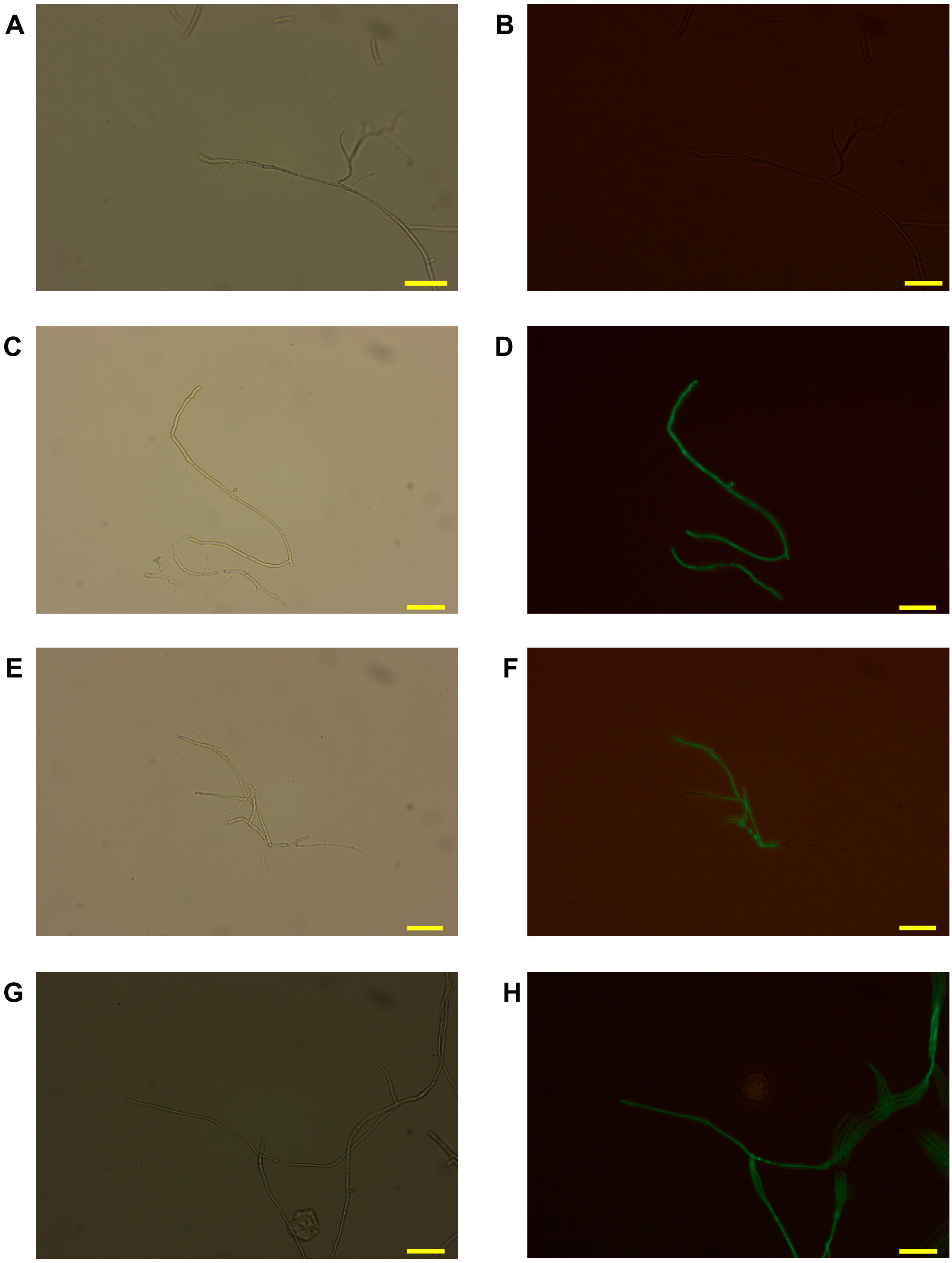

Supplemental Figure S1. Bright field (**A**,**C**,**E**,**G**) and fluorescent images (**B**,**D**,**F**,**H**) of WT (**A**,**B**), Tk-1 (**C**,**D**), Tk-3 (**E**,**F**), and Tk-19 (**G**,**H**). Images taken on Zeiss Axio Imager A2 Axiocam 105 with fluorescence excitation with scopeLED FLS140 at 470 nm. Bars show 500 μm.
