# Supplementary material for: Unanticipated Large-Scale Deletion in Fusarium graminearum Genome Using CRISPR/Cas9 and Its Impact on Growth and Virulence
Source: J Fungi (Basel). 2023 Jun 14;9(6):673. doi: 10.3390/jof9060673 (PMC10303880; doi:10.3390/jof9060673)
Supplement: Supplementary file 1 [file jof-09-00673-s001.zip › Table S1. Protoplast transformation media, buffers, and solutions (CRISPR paper).pdf]

Table S1. Protoplast transformation media, buffers, and solutions modified from (Guillemette et al. (2011).

Media Name	Components	Notes
Flask A	0.50 g Yeast extract<br>0.50 g Skim milk powder*<br>25mL Milli-Q (MQ) water	Autoclaved liquid cycle 20 min at 121°°C
Flask B	172.50 g Sucrose<br>250 mL MQ water	Autoclaved liquid cycle 20 min at 121°°C
Flask C	8.0g Granulated agar<br>225 mL MQ water	Autoclaved liquid cycle 20 min at 121°°C
1% Water Agar	5.0 g Granulated agar<br>500 mL MQ water	Autoclaved liquid cycle 20 min at 121°°C
Protoplasting Buffer	83 g $MgSO_4 \cdot H_2O$<br>500 mL MQ water	pH adjusted to 5.6<br>Autoclaved liquid cycle 20 min at 121°°C
40% (w/v) PEG-Calcium Transformation Solution	12 g PEG 3000<br>6.0 mL MQ water<br>7.5 mL 0.8M Mannitol (final 0.2M)<br>6.0 mL 0.5M $CaCl_2$ (final 0.1M)	0.22 μM bottle filtered
W5 Solution	0.093 g KCl (final 5mM)<br>4.6 g $CaCl_2 \cdot 2H_2O$ (final 125mM)<br>2.25 g NaCl (final 154mM)<br>7.8 g Glucose (final 177mM)<br>250 mL MQ water	0.22 μM bottle filtered
Protoplast Solution	10 mL Protoplasting buffer<br>200 mg Lysing enzyme (Sigma-Aldrich, St. Louis, MO, USA)<br>200 mg Yatalase (Takara Bio USA, Inc., San Jose, CA, USA)	0.45μM bottle filtered

* skim milk power used in place of casein
