# Supplementary figures and images for: Unanticipated Large-Scale Deletion in Fusarium graminearum Genome Using CRISPR/Cas9 and Its Impact on Growth and Virulence
Source: J Fungi (Basel). 2023 Jun 14;9(6):673. doi: 10.3390/jof9060673 (PMC10303880; doi:10.3390/jof9060673)

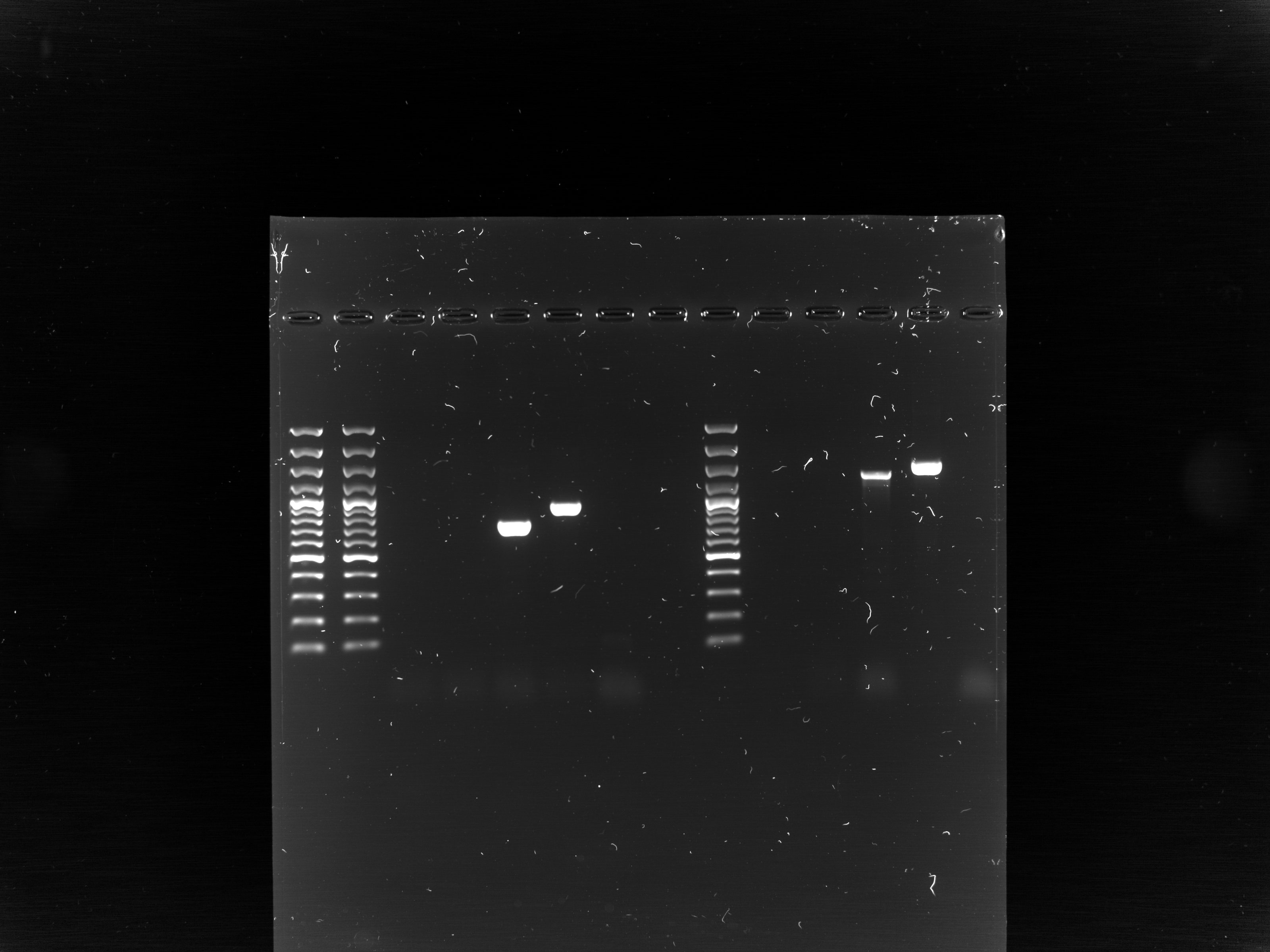

Supplement: Supplementary file 1 [file jof-09-00673-s001.zip › Supplementary File S2 Figure 2 Gel Images/2023-0527- gel 3_pub.tif]

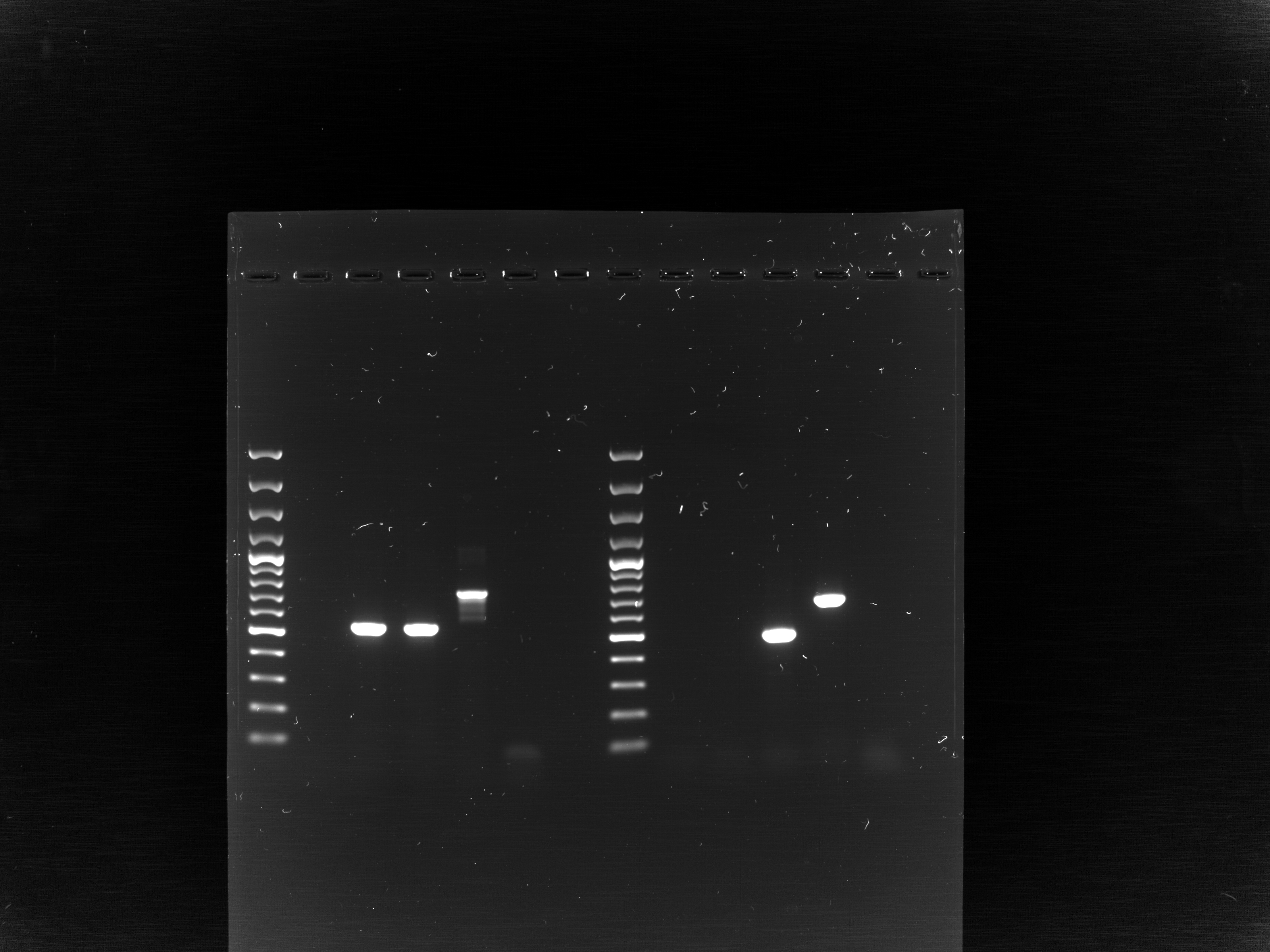

Supplement: Supplementary file 1 [file jof-09-00673-s001.zip › Supplementary File S2 Figure 2 Gel Images/gel 1a_pub.tif]
